# Increased circulating adiponectin is an independent disease activity marker in patients with rheumatoid arthritis: A cross-sectional study using the KURAMA database

**DOI:** 10.1371/journal.pone.0229998

**Published:** 2020-03-03

**Authors:** Hiroto Minamino, Masao Katsushima, Tamami Yoshida, Motomu Hashimoto, Yoshihito Fujita, Mirei Shirakashi, Wataru Yamamoto, Kosaku Murakami, Koichi Murata, Kohei Nishitani, Masao Tanaka, Hiromu Ito, Nobuya Inagaki, Shuichi Matsuda

**Affiliations:** 1 Department of Diabetes, Endocrinology and Nutrition, Graduate School of Medicine, Kyoto University, Kyoto, Japan; 2 Japan Society for the Promotion of Science, Tokyo, Japan; 3 Department of Rheumatology and Clinical Immunology, Graduate School of Medicine, Kyoto University, Kyoto, Japan; 4 Department of Nursing, Kyoto Prefectural University of Medicine, Kyoto, Japan; 5 Department of Advanced Medicine for Rheumatic Diseases, Graduate School of Medicine, Kyoto University, Kyoto, Japan; 6 Department of Health Information Management, Kurashiki Sweet Hospital, Kurashiki, Japan; 7 Department of Orthopedic Surgery, Graduate School of Medicine, Kyoto University, Kyoto, Japan; Ritsumeikan University, JAPAN

## Abstract

**Objective:**

To clarify the relationship among serum adiponectin, body composition, current disease activity and therapeutics of rheumatoid arthritis (RA).

**Methods:**

We conducted a cross-sectional study in RA patients under treatment with agents including biological disease-modifying antirheumatic drugs (bDMARDs) and Janus kinase (JAK) inhibitors. A total of 351 subjects from the Kyoto University RA Management Alliance cohort (KURAMA) were enrolled in the analysis. We classified the participants into five body composition groups according to the cut-off points for obesity and visceral fat used in Japan: body mass index (BMI), 18.5 kg/m^2^ for underweight and 25.0 kg/m^2^ for obesity, and visceral fat area (VFA), 100 cm^2^ for visceral adiposity.

**Results:**

Classification of body composition revealed that serum adiponectin levels and disease activity score (DAS28-ESR) in the low BMI group were significantly higher than those in the normal and overweight groups. Because both increased serum adiponectin and low BMI were previously reported as poor prognostic factors of RA, we performed multiple regression analysis to determine which factor was correlated with RA disease activity. Serum adiponectin level, but not BMI, was positively associated with DAS28-ESR (estimate = 0.0127, *p* = 0.0258). Subanalysis also showed that the use of bDMARD or JAK inhibitor did not have an obvious influence on circulating adiponectin.

**Conclusions:**

Classification of body composition and multiple regression analysis revealed a positive and independent correlation between serum adiponectin and DAS28-ESR in Japanese RA patients. Thus, serum adiponectin may be an important marker reflecting high disease activity of RA regardless of current medications.

## Introduction

Rheumatoid arthritis (RA) is a chronic autoimmune disease characterized by inflammatory destruction of joints. Cytokine and T cell signaling pathways are pivotal mediators of RA [1], and biological disease-modifying antirheumatic drugs (bDMARDs) and Janus kinase (JAK) inhibitors targeting the mediators have dramatically improved clinical outcomes [2, 3]. However, some patients continue to show insufficient response to several agents including the newer ones, and the patients may have an unknown contributor to sustained high disease activity.

Previous reports have revealed that low body mass index (BMI) is a poor prognostic factor of RA. Several studies have demonstrated that low BMI associates with radiographic progression and mortality [4–6], and that greater BMI is associated with lower risk of joint damage [7, 8]. These unexpected and beneficial results are called the “obesity paradox”, as adipose tissue is a potent source of pro-inflammatory cytokines such as tumor necrosis factor α (TNFα) and interleukin-6 (IL-6) [9]. The underlying mechanisms of joint inflammation among underweight patients are unclear and require further investigation.

Recently, both clinical and basic studies have reported a relationship between RA and adiponectin, a major adipokine secreted mainly from adipose tissue. Adiponectin possesses pleiotropic effects on inflammatory conditions of several chronic diseases. For example, it has anti-atherogenic and anti-inflammatory effects on metabolic traits such as type 2 diabetes, metabolic syndrome and cardiovascular disease [10–12]. However, conflicting effects on RA have been reported, such as that increased adiponectin in the synovium induces pro-inflammatory cytokines and matrix metalloproteinases (MMPs) [13, 14]. In clinical surveys on RA, some authors have shown a statistical association between serum adiponectin levels and radiographic joint destruction [15–17], but others have reported that hyperadiponectinemia does not correlate with disease activity score (DAS28) [18–20]. Furthermore, the influence of therapeutic agents (i.e., bDMARDs and corticosteroids) on circulating adiponectin remains contradictory [21–25].

As mentioned above, both low BMI and increased serum adiponectin have been reported as poor prognostic factors of RA. It is also known that serum adiponectin levels inversely correlate with BMI in general conditions and in patients with metabolic disorders including diabetes [26]. However, large-scale surveys of RA have not been done focusing on both BMI and serum adiponectin, and it is unclear which factor provides further contribution to RA disease activity. In addition, the effects of bDMARDs and JAK inhibitors on serum adiponectin levels are largely unknown. We performed a cross-sectional study to clarify the relationship among serum adiponectin, body composition, disease activity and therapeutic agents of RA patients.

## Material and methods

### Ethical statement (study setting)

In the current study, we recruited outpatients with RA from the KURAMA (Kyoto University Rheumatoid Arthritis Management Alliance) cohort. In brief, the KURAMA cohort was a prospective study that was established in May 2011 at the Center for Rheumatic Diseases in Kyoto University Hospital for the purpose of the proper control of RA and utilization of laboratory and clinical data for clinical investigations [27]. This study was designed under the principals of the Declaration Helsinki and approved by the Medical Ethics Committee of Kyoto University Graduate School and Faculty of Medicine (Approval number: R0357). All participants provided written informed consent.

### Study population and design

All outpatients who were over 18 years old and fulfilled the 2010 American College of Rheumatology (ACR)/European League against Rheumatism (EULAR) classification criteria were included between 1^st^ May and 30^th^ November 2016 (n = 441) [28]. After excluding 55 patients without a complete data set, using an electronic device, fracture, pain, abdominal surgery or pregnancy, which prevented measurement of body composition, and 35 with confounding conditions or treatments such as dialysis treatment, hepatitis, sex-hormone suppression or replacement therapy, psychiatric disorders and cognitive impairment, the remaining 351 patients were enrolled in this study.

### Evaluation of serum adiponectin concentrations and RA-related factors

Total serum adiponectin level was measured using latex particle-enhanced turbidimetric immunoassay (LTIA) (SRL, Inc. Tokyo, Japan). We assessed disease activity and physical disability of RA using the 28-Joint RA Disease Activity Score (DAS28-ESR) and the health assessment questionnaire-disability index (HAQ), respectively. Baseline values of serological data were evaluated including rheumatoid factor (RF), matrix metalloproteinase-3 (MMP-3), anti-cyclic citrullinated peptide (anti-CCP antibody), C-reactive protein (CRP), erythrocyte sedimentation rate (ESR), estimated glomerular filtration (eGFR), hemoglobin (Hb) and albumin (Alb). We also reviewed the use of therapeutic agents including methotrexate (MTX), prednisolone (PSL), TNF inhibitors (infliximab, adalimumab, etanercept, certolizumab pegol, golimumab), IL-6 receptor inhibitor (tocilizumab), cytotoxic T-lymphocyte antigen 4-immunoglobulin (abatacept), and JAK inhibitor (tofacitinib). The only targeted synthetic DMARDs (tsDMARDs) used in the cohort were tofacitinib, which was included in the biological agent category for statistical evaluation.

### Data collection and classification of body composition

We measured height, weight, waist circumference (WC), and indicators of body composition including visceral fat area (VFA), subcutaneous fat area (SFA) and skeletal muscle ratio (SMR) with standardized protocols, as previously described [29].

Briefly, WC was measured at the navel position with a non-stretchable tape while standing. VFA and SFA were measured with a dual bioelectrical impedance analyzer (HDS-2000 DUALSCAN, Omron Healthcare Co., Japan), and the skeletal muscle mass was assessed by a Z impedance analyzer (HBF-701 KARADASCAN, Omron Healthcare Co., Japan) [30]. SMR was calculated by dividing the skeletal muscle mass by body weight. With these indicators, we classified RA outpatients into five body composition groups according to the criteria for obesity and visceral fat in Japan [31]. Specifically, we divided RA outpatients using cut-off points of BMI (18.5 kg/m^2^ for underweight and 25.0 kg/m^2^ for obesity) and VFA (100 cm^2^ for visceral adiposity).

### Statistical analysis

Data were described as the mean ± standard deviation (SD) for continuous variables and numbers (%) for categorical variables. Differences in body composition groups and comparisons of adiponectin levels among types of treatments were assessed by a Steel-Dwass test or one-way analysis of variance (ANOVA) for continuous variables, or Fisher’s exact test for categorical variables. To analyze effects of serum adiponectin levels on RA disease activity (DAS28-ESR), we adopted a multiple standardized linear regression model with identity link and exponential distribution including demographic factors (age, sex, body mass index, VFA/SFA ratio), RA-related-factors (RA duration, RF, anti-CCP antibody and presence or absence of biological agents, MTX and PSL), life style-related factors (diabetes mellitus, hypertension, dyslipidemia and smoking habit) and serological factors (adiponectin, eGFR). In subgroup analysis according to sex, we selected variables for multiple regression analysis including RF, anti-CCP antibody, age, body mass index and adiponectin. We included possible covariates previously reported in the analysis, and eliminated multicollinearity as detected by variance inflation factor >10. All statistical analyses were performed by JMP^®^ 14 (SAS Institute Inc., Cary, NC, USA) and P values < 0.05 were considered significant.

## Results

### Baseline characteristics of RA outpatients in this study

The included data of 351 patients (291 women, 60 men) were subjected to the following analysis. The baseline characteristics are described in Table 1. The average age was 61.8 years and the average RA disease duration was 10.6 years. BMI and DAS28-ESR of the enrolled patients were generally low compared to those in previous reports. [16, 32] In current therapeutics, usage rates of MTX, bDMARDs, and PSL are 72.6, 52.4, and 20.8% respectively, and there are newer biological agents including anti-TNFα treatments and anti-IL-6 treatments.

**Table 1:** Basic demographical and clinical characteristics of study population.

			RA patients
Items			(*N* = 351)
			
Age, years			61.8 ± 12.0
Male, *n* (%)			60.0 (17.1)
Body composition parameters			
	BMI, kg/m^2^		22.6 ± 3.7
	VFA, cm^2^		62.0 ± 32.5
	SFA, cm^2^		153.6 ± 69.9
	V/S ratio		0.43 ± 0.19
Systemic skeletal muscle ratio, %			24.6 ± 3.2
Body fat percentage, %			
	Male		24.8 ± 5.6
	Female		32.1 ± 5.0
Waist circumference, cm			84.2 ± 10.2
Laboratory data			
	Hemoglobin, g/dL		12.8 ± 1.5
	Albumin, g/dL		4.03 ± 0.30
	eGFR, ml/min/1.73m^2^		74.5 ± 18.1
	Adiponectin, μg/mL		14.8 ± 8.5
Comorbidities			
	Hypertension, *n* (%)		113 (32.2)
	Diabetes Mellitus, *n* (%)		29 (8.3)
	Dyslipidemia, *n* (%)		130 (37.0)
	Smoking habit, *n* (%)		29 (8.2)
RA disease characteristics			
	Duration, years		10.6 ± 9.5
	RF, IU/mL		39.2 (8–2833.6)
	MMP-3, ng/mL		56.8 (18.2–633.6)
	Anti-CCP antibody, U/mL		51 (0.6–3260)
	CRP, mg/dL		0.1 (0.1–9.6)
	DAS28-ESR		2.52 (0.33–7.20)
	HAQ score		0.29 (0–2.50)
	Stage*		2.40 ± 1.16
	Class*		1.52 ± 0.59
Current RA therapeutics			
	MTX use, *n* (%)		255 (72.6)
	Dose of MTX use, mg/day (mean of users)		8 (2–16)
	Other cs DMARDs use, *n* (%)		46 (13.1)
	ts DMARDs use, *n* (%)		2 (1.0)
	Prednisoline use, *n* (%)		73 (20.8)
	Dose of Prednisolone use, mg/day (mean of users)		4 (1–10)
	Biological agent use, *n* (%)		184 (52.4)
		Abatacept, *n*	38
		Adalimumab, *n*	14
		Certolizumab pegol, *n*	10
		Etanercept, *n*	23
		Golimumab, *n*	26
		Infliximab, *n*	27
		Tocilizumab, *n*	44
		Tofacitinib, *n*	2

Continuous variable data are presented as mean (± SD), and categorical variables are shown as numbers (%). Data of RA characteristics and current therapeutics are expressed as median (range).

*RA* rheumatoid arthritis, *BMI* body mass index, *VFA* visceral fat area, *SFA* subcutaneous fat area, *V/S* ratio visceral/subcutaneous fat area, *eGFR* estimated glomerular filtration, *RF* rheumatoid factor, *MMP-3* matrix metalloproteinase 3, *anti-CCP antibody* anti-cyclic citrullinated peptide antibody, *CRP* C-reactive protein, *DAS28-ESR* 28-joint disease activity score using erythrocyte sedimentation rate, *HAQ* health assessment questionnaire, *MTX* methotrexate, *csDMARD* conventional synthetic disease modifying anti-rheumatic drugs, *tsDMARD* targeted synthetic DMARD

*Steinbrocker's stage and class

### Serum adiponectin levels and RA disease activity are highest in the low BMI group

Next, to understand whether body composition of RA patients affects RA disease activity and serum adiponectin levels, we divided the 351 patients into five body composition phenotypes: underweight (BMI < 18.5, VFA < 100), normal weight (+) visceral adiposity (-) (18.5 ≤ BMI < 25, VFA < 100), normal weight (+) visceral adiposity (+) (18.5 ≤ BMI < 25, VFA ≥ 100), overweight (+) visceral adiposity (-) (25 < BMI, VFA < 100), and overweight (+) visceral adiposity (+) (25 < BMI, VFA ≥ 100) (Table 2). No significant differences were detected in age, RA-duration, or current RA medications among the various body composition groups. In contrast, both serum adiponectin levels and DAS28-ESR were significantly higher only in the underweight group compared to the others (mean ± SD, 20.9 ± 12.5, *p* = 0.017 for adiponectin, and mean ± SD, 3.04 ± 1.03, *p* < 0.001 for DAS28-ESR). Hence, RA patients with low body weight had remarkably higher serum adiponectin levels along with higher RA disease activity than those with normal weight or obesity.

**Table 2:** Baseline demographics of RA patients stratified by body composition phenotype (VFA and BMI).

		Underweight	Normal weight		Overweight		
		BMI < 18.5	18.5 ≤ BMI < 25		BMI ≥ 25		*p* value *
		VFA < 100	VFA < 100	VFA ≥ 100	VFA < 100	VFA ≥ 100	
	Visceral adiposity	(-)	(-)	(+)	(-)	(+)	
*(N* = 351)		*n* = 37 (10.5%)	*n* = 223 (63.5%)	*n* = 12 (3.4%)	*n* = 43 (12.3%)	*n* = 36 (10.3%)	
Age, year		61.3 ± 13.3	61.2 ± 12.2	71.8 ± 6.7	61.7 ± 11.6	62.9 ± 9.8	0.0501
Sex, n (Male/Female)		1/36	31/192	7/5	5/38	16/20	< 0.001
Body composition parameters							
	VFA, cm^2^	33.7 ± 15.0	50.7 ± 19.1	113.8 ± 13.6	77.1 ± 15.2	125.5 ± 26.4	< 0.001
	SFA, cm^2^	69.6 ± 25.7	138.0 ± 47.9	156.9 ± 39.8	230.7 ± 57.3	244.0± 67.1	< 0.001
	V/S ratio	0.68 ± 0.47	0.40 ± 0.17	0.76 ± 0.16	0.35 ± 0.12	0.55 ± 0.16	< 0.001
Systemic skeletal muscle ratio, %		25.5 ± 3.3	24.8 ± 3.2	25.8 ± 3.3	23.1 ± 3.4	24.6 ± 3.5	0.006
Waist circumference, cm							
	Male	63.0 ± 0.0	83.4 ± 6.0	87.9 ± 3.9	90.9 ± 6.7	98.1 ± 5.5	< 0.001
	Female	71.0 ± 5.1	81.5 ± 7.3	92.4 ± 4.5	94.0 ± 7.2	100.0 ± 6.9	< 0.001
Laboratory data							
	Hemoglobin, g/dL	12.2 ± 1.5	12.6 ± 1.4	13.2 ± 1.2	13.4 ± 1.6	13.8 ± 1.6	< 0.001
	Albumin, g/dL	3.98 ± 0.35	4.02 ± 0.31	4.05 ± 0.20	4.04 ± 0.26	4.08 ± 0.31	0.762
	eGFR, ml/min/1.73m^2^	79.2 ± 22.4	74.7 ± 17.8	70.3 ± 13.3	73.1 ± 17.1	72.0 ± 17.1	0.386
	Adiponectin, μg/mL	20.9 ± 12.5**	14.8 ± 7.9	12.3 ± 6.1	11.8 ± 5.9	12.8 ± 7.5	< 0.001
RA disease characteristics							
	Disease duration, year	11.1 ± 7.8	10.5 ± 10.0	11.0 ± 8.7	12.0 ± 10.3	9.0 ± 7.1	0.716
	HAQ score	0.66 ± 0.62	0.45 ± 0.55	0.69 ± 0.69	0.48 ± 0.60	0.51 ± 0.59	0.229
	CRP, mg/dl	0.66 ± 1.81	0.33 ± 0.75	0.33 ± 0.38	0.26 ± 0.33	0.39 ± 0.54	0.243
	DAS28-ESR	**3.04 ± 1.03**^§^	2.60 ± 0.90	2.84 ± 0.87	2.72 ± 0.98	2.32 ± 0.98	0.017
Current therapeutic agent							
	csDMARD use, n (%)	32 (86.5)	196 (87.9)	9 (75.0)	35 (81.4)	29 (80.1)	0.4886
	tsDMARD use, n (%)	0 (0)	2 (0.9)	0 (0)	0 (0)	0 (0)	0.887
	Biological agent use, n (%)	21 (56.8)	114 (51.1)	6 (50.0)	22 (51.2)	19 (52.8)	0.979
	Prednisolone use, n (%)	9 (24.3)	36 (16.1)	4 (33.3)	13 (30.2)	11 (30.6)	0.067

Data are expressed as mean ± SD, or number (%) *BMI* body mass index, *VFA* visceral fat area, *SFA* subcutaneous fat, *V/S* visceral/subcutaneous fat area, *eGFR* estimated glomerular filtration, *HAQ* health assessment questionnaire, *CRP* C-reactive protein, *DAS28-ESR* disease activity score in 28 joints using erythrocyte sedimentation rate, *csDMARD* conventional synthetic disease modifying anti-rheumatic drugs, *tsDMARD* targeted synthetic DMARD

*p-values for analysis of variance (ANOVA) for continuous variables or Fisher’s exact test for categorical variables

**p < 0.05 for multiple comparisons using Steel–Dwass test with the group of normal weight/visceral adiposity (-), overweight/visceral adiposity (-) and overweight/visceral adiposity (+), p = 0.028, p = 0.003, p = 0.028, respectively

^§^p < 0.05 for multiple comparisons using Steel–Dwass test with normal weight/visceral adiposity (-) and overweight/visceral adiposity (+), p = 0.041, p = 0.019, respectively

### Serum adiponectin levels rather than BMI may be independently associated with DAS28-ESR

Previous studies had reported that serum adiponectin levels were negatively correlated to BMI in general conditions, and that both increased serum adiponectin and low BMI were poor prognostic factors of RA progression. To determine whether serum adiponectin levels were independently related with RA disease activity, we performed multiple regression analysis. With DAS28-ESR as the dependent variable, serum adiponectin level, but not BMI, was significantly associated with higher DAS28-ESR (Table 3). In addition, age, PSL use, RF, anti-CCP antibody and eGFR were positively associated, and male sex was negatively associated with DAS28-ESR as independent variables. In subgroup analysis based on sex differences, we also found that serum adiponectin level was independently correlated with DAS28-ESR in female patients as well as in male ones (S1 Table). These results suggest that the serum adiponectin level may be an important parameter in terms of assessment of RA disease state.

**Table 3:** Multiple regression analysis for independent factors associated with DAS28-ESR.

Dependent variables		Independent variables					95%CI	
				Estimates	Std. Error	*p-*value	Lower	Upper
DAS28-ESR		Prednisolone (+)		0.553	0.117	< .0001	0.323	0.782
		RF (1 IU/mL)		0.0007	0.00001	< .0001	0.00028	0.00099
		Age (10 years)		0.171	0.049	< .0001	0.075	0.27
		eGFR (10 ml/min/1.73m^2^)		0.083	0.028	0.0033	0.028	0.14
		Sex (male)		-0.406	0.14	0.0037	-0.68	-0.13
		Adiponectin (1 μg/mL)		0.0127	0.0057	0.0258	0.0015	0.024
		Anti-CCP antibody (10 U/mL)		0.0025	0.0011	0.0259	0.0003	0.0047

Covariates were selected from demographic, RA activity-related and life style-related factors: age, sex, body mass index, V/S ratio, eGFR, RA duration, RF, anti-CCP antibody, biological agent use, MTX use, PSL use, diabetes mellitus, hypertension, dyslipidemia, smoking habit and adiponectin. Units for estimates values are expressed in units in parentheses.

*RF* rheumatoid factor, *eGFR* estimated glomerular filtration rate, *anti-CCP antibody* anti-cyclic citrullinated peptide antibody.

### Circulating adiponectin levels are not affected by biological agents

**Fig 1:**
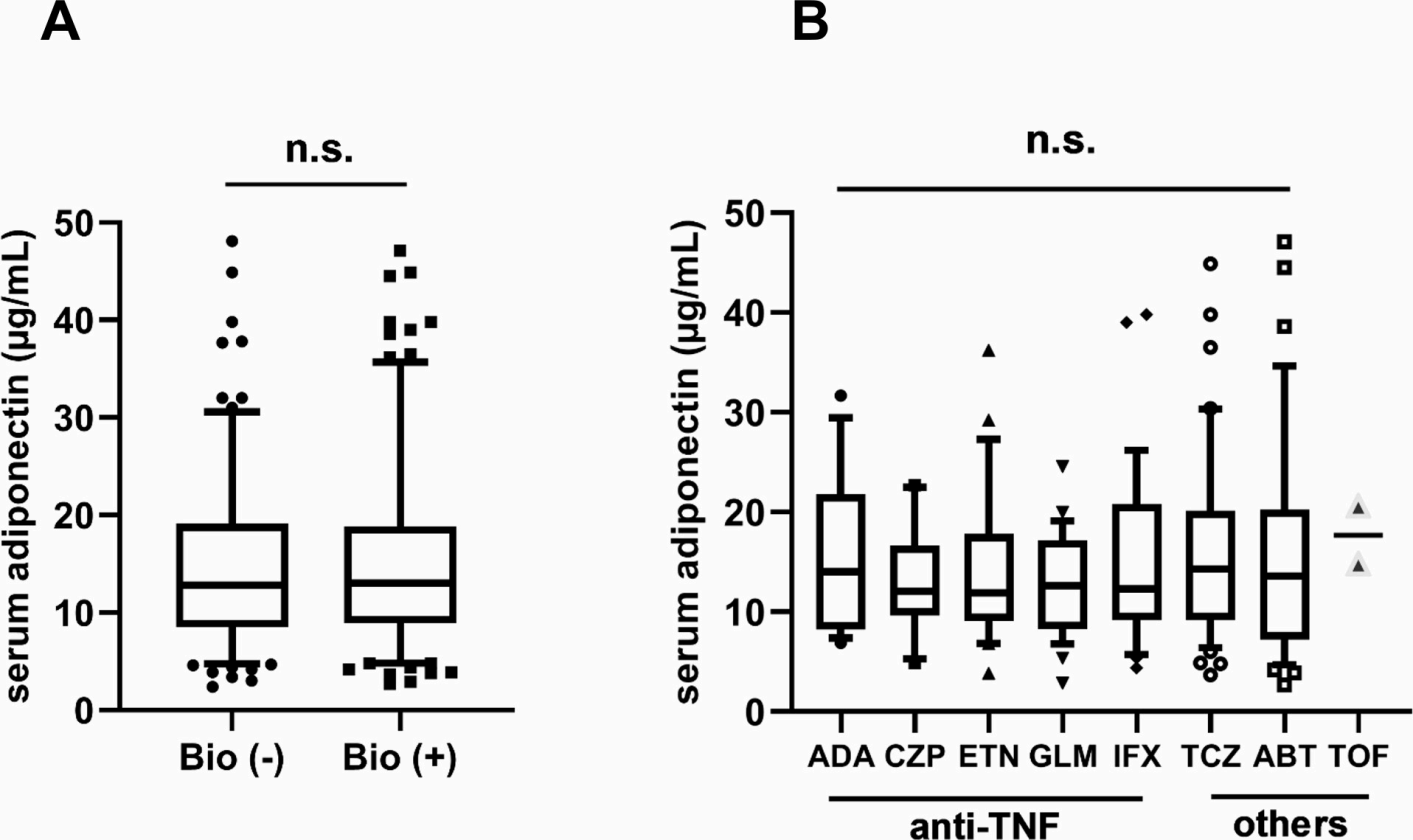
**Box-and-whisker plots of serum adiponectin levels for subjects by use of biological agents (A) and type of biological agent (B).** Boxplots show IQR from the 1^st^ quartile to the 3^rd^ quartile. Whiskers are drawn to the 5^th^ percentile and 95^th^ percentile point. Mean serum adiponectin levels did not differ significantly between users of biological agents and non-users (A), or among kinds of biological agent (B). *Bio* biological agents, *ABT* abatacept, *ADA* adalimumab, *CZP* certolizumab pegol, *ETN* etanercept, *GLM* golimumab, *IFX* infliximab, *TCZ* tocilizumab, *TOF* tofacitinib, *TNF* tumor necrosis factor, *N*.*S*. not significant.

In addition, we assessed the influence of bDMARDs and JAK inhibitor on serum adiponectin levels. Fig 1 shows the histogram of serum adiponectin levels of patients divided into each treatment group. Although previous studies have shown that serum adiponectin levels are affected by RA therapeutics such as anti-TNFα treatments [22, 25], our data indicate that biological agents and JAK inhibitor do not significantly affect serum adiponectin levels.

## Discussion

In order to evaluate the relationship among serum adiponectin, body composition and RA disease activity, we performed a cross-sectional survey of a Japanese RA population under treatment. Classification of body composition revealed that DAS28-ESR and serum adiponectin levels were significantly increased only in the underweight group (BMI < 18.5, VFA < 100). Accounting for covariates including BMI, serology and therapeutics, multiple regression analysis showed a positive correlation between DAS28-ESR and serum adiponectin. These results correspond to previous findings that patients with lower BMI have exacerbated disease progression [5, 6], and demonstrate that serum adiponectin, rather than BMI, may be a main contributor to the disease activity regardless of current medication.

In addition to serum adiponectin, other independent variables were also associated with DAS28-ESR in multiple regression analysis, such as PSL use, RF, anti-CCP antibody, age, sex and eGFR (Table 3). The long-term use of PSL potentially has multiple adverse effects, and its use is usually limited to refractory patients with high disease activity according to clinical recommendations [3]. Previous studies have also reported that high titers of RF or anti-CCP antibody are poor prognostic factors [33], and that greater age and female sex are independently associated with the increased ESR levels in RA patients [34]. Furthermore, eGFR levels are generally overestimated in lean populations, and thus its levels may be highest in the underweight group with high disease activity (Table 2).

There was no significant difference of serum adiponectin level between normal weight and overweight in the body composition analysis, although its level inversely correlated with BMI and VFA in the whole population of our study (data not shown) as well as in previous studies [35, 36]. These findings may be attributed to several reasons. First, the relationship between serum adiponectin level and BMI/VFA shows exponential decay as previously reported [11], and thus the difference of its level gets smaller according to weight gain. Second, the number of subjects in each body composition is unbalance and might not be enough to show the statistically significant difference.

Subanalysis of therapeutic agents indicates that they do not have an obvious influence on circulating adiponectin. Other biomarkers such as MMP-3, TNFα, IL-6 and CRP are more or less interfered with by PSL, TNF inhibitors and IL-6 inhibitors, and may not necessarily reflect disease activity states of RA. Even though further longitudinal studies are needed, serum adiponectin seems to be a novel activity marker regardless of therapeutic agents.

Although it has been generally accepted that adiponectin has an anti-atherogenic and anti-inflammatory effect, recent clinical and basic research has indicated a deleterious role of adiponectin under inflammatory conditions. Epidemiological studies have reported a positive association between circulating adiponectin and all-cause mortality on several chronic diseases such as cardiovascular disease, type 2 diabetes and chronic kidney disease [37]. Another survey on RA has revealed that serum adiponectin significantly correlates with radiographic damage, but that other adipokines (resistin and leptin) do not [16]. Furthermore, *in vitro* studies have shown that adiponectin induces MMP-3, IL-6 and monocyte chemotactic protein-1 (MCP-1) expression in human chondrocytes [13, 38]. Klaus et al. have also proved that adiponectin promotes higher-yield secretion of chemical mediators (i.e., MCP-1, IL-6, IL-8 and MMP-3) from RA synovial fibroblasts compared to those in osteoarthritis, and that it induces lymphocytes to synthesize TNFα, IL-6 and IL-8 [39]. These results suggest that adiponectin plays a potential pathophysiological role in RA.

**Fig 2:**
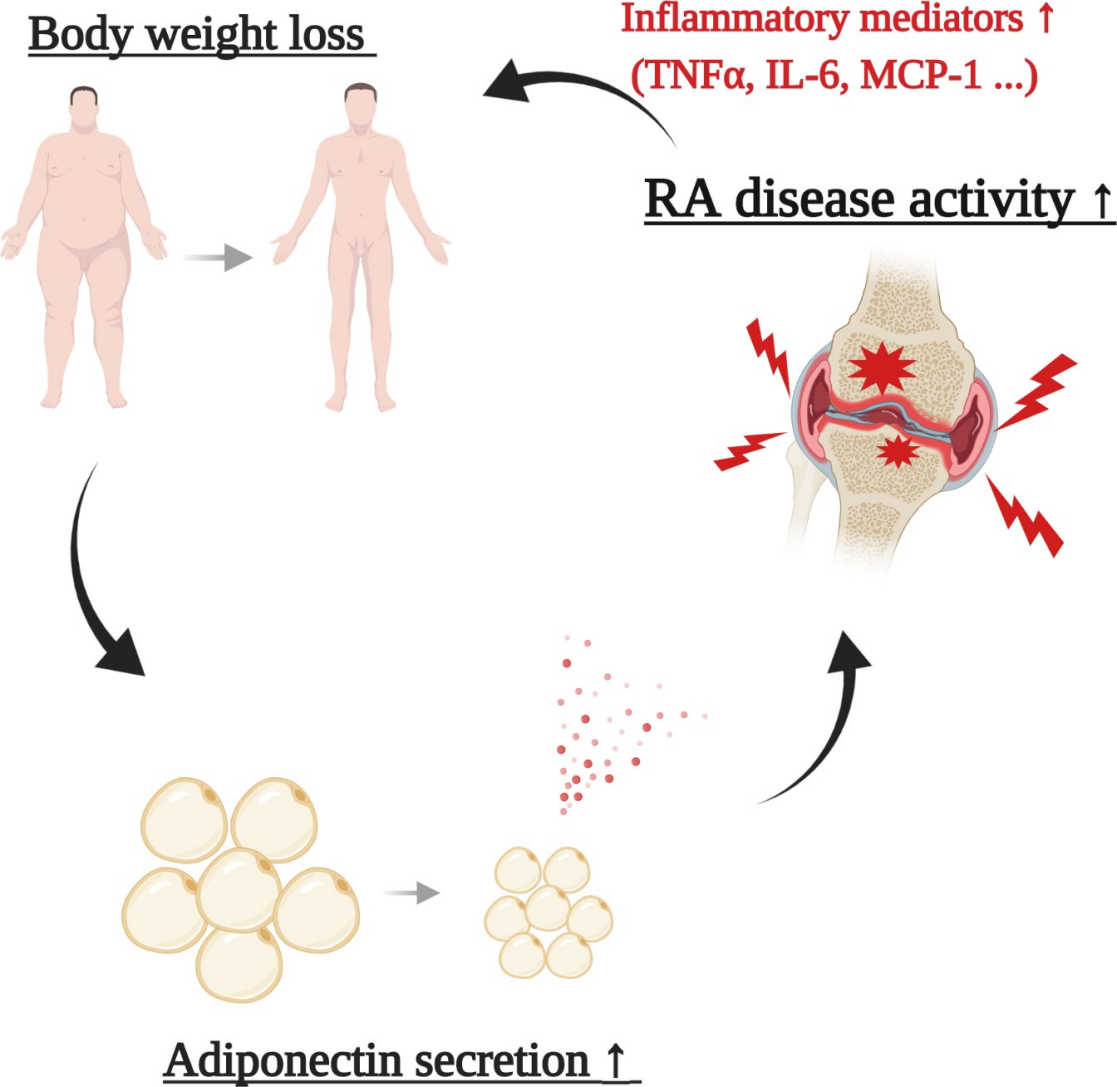
A proposed cycle model of the exacerbation of rheumatoid arthritis in terms of adiponectin. Hyperadiponectinemia enhances inflammation and cytokine/chemokine production in RA synovial joints. These inflammatory mediators induce loss of body weight including white adipose tissue, and additional adiponectin is secreted from the remaining white adipose tissue. This cycle of exacerbation may occur especially in RA patients having low body weight. *TNF* tumor necrosis factor, *IL-6* interleuin-6, *MCP-1* Monocyte Chemotactic Protein-1.

In the present study, the classification of body composition revealed the relationship among BMI, serum adiponectin and RA disease activity. Using the same classification method, our group previously showed that RA patients with low BMI had minimal scores of carotid atherosclerosis [29]. These results indicate that underweight RA have the highest disease activity and minimal atherogenic change. It seems to implicate a biphasic mediator in its pathogenesis; that is, hyperadiponectinemia may protect against systemic atherosclerosis but drive cytokine production from affected joints, resulting in body weight loss followed by additional secretion of circulating adiponectin (Fig 2).

There are several notable limitations in our study. This is a cross-sectional clinical study without longitudinal data, and our results do not imply causation. The long-term influence of each bDMARD or JAK inhibitor on serum adiponectin is still unknown, and the pathophysiological role of adiponectin in RA progression needs further investigation including *in vitro* studies. In our university hospital, the frequency of bDMARDs use exceeds 50% and might differ from that in other medical institutions. It is unclear whether our findings can be generalized to any other population, because Japanese have the lowest average BMI among developed countries, and because more than half of our participants is in one of the five groups (normal weight and VFA<100). Finally, there might be unconsidered covariates affecting RA severity or serum adiponectin such as genetic variants (i.e., RA risk HLA alleles, and SNPs in adiponectin genes) [40, 41].

In conclusion, classification of body composition and multiple regression analysis showed a positive and independent correlation between serum adiponectin and DAS28-ESR, and biological agents did not affect serum adiponectin levels. Therefore, measurement of serum adiponectin may be potentially useful for assessing disease activity of RA regardless of current medications.

## Supporting information

S1 Table Multiple regression analysis for independent factors associated with DAS28-ESR by sex differences. Subgroup analysis based on women (S1A Table) and men (S1B Table) was performed. Covariates were selected from RF, anti-CCP antibody, age, BMI and adiponectin, Units for estimates values are described in units in parentheses. *RF* rheumatoid factor, *anti-CCP antibody* anti-cyclic citrullinated peptide antibody, *BMI* body mass index.
(DOCX)

S2 Table Multiple regression analysis for factors associated with DAS28-ESR. Covariates were selected from demographic, RA activity-related and life style-related factors: age, sex, body mass index, V/S ratio, eGFR, RA duration, RF, anti-CCP antibody, biological agent use, MTX use, PSL use, diabetes mellitus, hypertension, dyslipidemia, smoking habit and adiponectin. Units for estimates values are expressed in units in parentheses. RF rheumatoid factor, eGFR estimated glomerular filtration rate, anti-CCP antibody anti-cyclic citrullinated peptide antibody, MTX Methotrexate, RA rheumatoid arthritis, BMI body mass index.
(DOCX)
