# Supplementary material for: Increased circulating adiponectin is an independent disease activity marker in patients with rheumatoid arthritis: A cross-sectional study using the KURAMA database
Source: PLoS One. 2020 Mar 3;15(3):e0229998. doi: 10.1371/journal.pone.0229998 (PMC7053773; doi:10.1371/journal.pone.0229998)
Supplement: S1 Table — Subgroup analysis based on women (S1A Table) and men (S1B Table) was performed. Covariates were selected from RF, anti-CCP antibody, age, BMI and adiponectin, Units for estimates values are described in units in parentheses. RF rheumatoid factor, anti-CCP antibody anti-cyclic citrullinated peptide antibody, BMI body mass index. (DOCX) [file pone.0229998.s001.docx]

**S1 Table　Multiple regression analysis for independent factors associated with DAS28-ESR by sex differences**

**S1A Table**

| **Dependent variables** | | **Independent variables** | |  |  |  | **95%CI** | |
| --- | --- | --- | --- | --- | --- | --- | --- | --- |
| **(Female n = 291 )** | |  |  | **Estimates** | **Std. Error** | ***p-*value** | **Lower** | **Upper** |
| DAS28-ESR | | RF (1 IU/mL) |  | 0.001 | 0.256 | < .0001 | 0.00065 | 0.0017 |
|  | | age (10 years) |  | 0.123 | 0.0043 | 0.0048 | 0.0038 | 0.021 |
|  | | Adiponectin (1 µg/mL) | | 0.013 | 0.0062 | 0.038 | 0.00072 | 0.025 |
|  |  | Anti-CCP antibody (10 U/mL) | | 0.00019 | 0.00013 | 0.167 | -0.000078 | 0.00049 |
|  | | BMI |  | 0.0002 | 0.00026 | 0.989 | -0.028 | 0.029 |

| **Dependent variables** | | **Independent variables** | |  |  |  | **95%CI** | |
| --- | --- | --- | --- | --- | --- | --- | --- | --- |
| **(Male n = 60 )** | |  |  | **Estimates** | **Std. Error** | ***p-*value** | **Lower** | **Upper** |
| DAS28-ESR | | RF (1 IU/mL) |  | 0.00057 | 0.00025 | 0.024 | 0.000078 | 0.0011 |
|  | | BMI |  | -0.078 | 0.035 | 0.031 | -0.148 | -0.0075 |
|  | | Adiponectin (1 µg/mL) | | 0.038 | 0.019 | 0.0498 | 0.000038 | 0.075 |
|  |  | age (10 years) |  | 0.166 | 0.01 | 0.113 | -0.04 | 0.037 |
|  |  | Anti-CCP antibody (10 U/mL) | | 0.00028 | 0.00023 | 0.244 | -0.00019 | 0.00073 |

**S1B Table**

**（S1 Table Legend）**Subgroup analysis based on women (S1A Table) and men (S1B Table) was performed. Covariates were selected from RF, anti-CCP antibody, age, BMI and adiponectin, Units for estimates values are described in units in parentheses

*RF* rheumatoid factor, *anti-CCP antibody* anti-cyclic citrullinated peptide antibody, *BMI* body mass index
