# Supplementary material for: Increased circulating adiponectin is an independent disease activity marker in patients with rheumatoid arthritis: A cross-sectional study using the KURAMA database
Source: PLoS One. 2020 Mar 3;15(3):e0229998. doi: 10.1371/journal.pone.0229998 (PMC7053773; doi:10.1371/journal.pone.0229998)
Supplement: S2 Table — Covariates were selected from demographic, RA activity-related and life style-related factors: age, sex, body mass index, V/S ratio, eGFR, RA duration, RF, anti-CCP antibody, biological agent use, MTX use, PSL use, diabetes mellitus, hypertension, dyslipidemia, smoking habit and adiponectin. Units for estimates values are expressed in units in parentheses. RF rheumatoid factor, eGFR estimated glomerular filtration rate, anti-CCP antibody anti-cyclic citrullinated peptide antibody, MTX Methotrexate, RA rheumatoid arthritis, BMI body mass index. (DOCX) [file pone.0229998.s002.docx]

| **Dependent variables** | **variables** |  |  |  | **95%CI** | |
| --- | --- | --- | --- | --- | --- | --- |
|  |  | **Estimates** | **Std. Error** | ***p-*value** | **Lower** | **Upper** |
| DAS28-ESR | Predonisolone (+) | 0.553 | 0.117 | < .0001 | 0.323 | 0.782 |
|  | RF (1 IU/mL) | 0.0007 | 0.00001 | < .0001 | 0.00028 | 0.00099 |
|  | age (10 years) | 0.171 | 0.049 | < .0001 | 0.075 | 0.27 |
|  | eGFR (10 ml/min/1.73m²) | 0.083 | 0.028 | 0.0033 | 0.028 | 0.14 |
|  | Sex (male) | -0.406 | 0.14 | 0.0037 | -0.68 | -0.13 |
|  | Adiponectin (1 µg/mL) | 0.0127 | 0.0057 | 0.0258 | 0.0015 | 0.024 |
|  | Anti-CCP antibody (10 U/mL) | 0.0025 | 0.0011 | 0.0259 | 0.0003 | 0.0047 |
|  | Hypertension (+) | -0.071 | 0.111 | 0.52 | -0.29 | 0.15 |
|  | Diabetes mellitus (+) | 0.32 | 0.17 | 0.06 | -0.014 | 0.66 |
|  | Dyslipidemia (+) | -0.134 | 0.097 | 0.17 | 0.33 | 0.057 |
|  | Biological agent (+) | -0.053 | 0.09 | 0.55 | -0.23 | 0.12 |
|  | MTX (+) | 0.12 | 0.1 | 0.28 | -0.092 | 0.324 |
|  | Smoking (+) | -0.15 | 0.1 | 0.13 | -0.358 | 0.048 |
|  | V/S ratio | 0.035 | 0.253 | 0.89 | -0.46 | 0.53 |
|  | RA duration (1 year) | 0.0074 | 0.005 | 0.14 | -0.0025 | 0.0173 |
|  | BMI | -0.0073 | 0.014 | 0.6 | -0.034 | 0.02 |

**S2 Table. Multiple regression analysis for factors associated with DAS28-ESR**

**（S2 Table Legend）**Covariates were selected from demographic, RA activity-related and life style-related factors: age, sex, body mass index, V/S ratio, eGFR, RA duration, RF, anti-CCP antibody, biological agent use, MTX use, PSL use, diabetes mellitus, hypertension, dyslipidemia, smoking habit and adiponectin. Units for estimates values are expressed in units in parentheses

*RF* rheumatoid factor, *eGFR* estimated glomerular filtration rate, *anti-CCP antibody* anti-cyclic citrullinated peptide antibody, *MTX* Methotrexate, *RA* rheumatoid arthritis, *BMI* body mass index.
